# Appointments in the University of Louisville

**Published:** 1852-09

**Authors:** 


					﻿Drs. Austin Flint and B. Palmer, late of Buffalo Medical College,
liave accepted chairs in the University of Louisville.
				

